# Supplementary material for: Defining harmful news reporting on community firearm violence: A modified Delphi consensus study
Source: PLoS One. 2024 Dec 18;19(12):e0316026. doi: 10.1371/journal.pone.0316026 (PMC11654925; doi:10.1371/journal.pone.0316026)
Supplement: S1 Appendix — (PDF) [file pone.0316026.s001.pdf]

Delphi Consensus on Harmful News Reporting on Community Firearm Violence: Round 1

Welcome Delphi Panelists!

This is a Temple University research study conducted by Jessica Beard, MD, MPH and her research team. Your participation in this research study is voluntary. The purpose of this study is to understand the perspectives of experts like you on what constitutes harmful news reporting on firearm violence in Philadelphia. The group of experts in this study is called a Delphi Panel. You have been invited to participate as an expert in this Delphi Panel because you are one or more of the following: a survivor or co-victim with lived experience of firearm violence, a journalist, and/or an academic expert in this area.

If you agree to participate, you will take part in three rounds of surveys over the course of six months. Each survey will take approximately 30 minutes to complete. No personally identifiable information will be recorded about you, and the investigators will not attempt to re-identify any of your information. However, there is a possibility that researchers could determine your identity based on the demographic information you provide because the participant pool is relatively small. This means there is a small risk of loss of your confidentiality. Your de-identified data will be kept for use in future research and shared with other researchers. Your responses to the survey will be combined with those of the other panelists and summarized in a report to further protect your anonymity.

Upon completion of each survey, you will receive $75 to compensate you for your time. This means that if you complete all three surveys, you will receive a total of $225. Payments will be made to you using ClinCard, a secure, reloadable MasterCard debit card supported by Greenphire. You may use this card online or at any store that accepts MasterCard. We will mail you the card. You will be given one card for the entire time of your participation, which may be used again if you participate in the subsequent surveys. Be sure to read the information included with your ClinCard, including the cardholder agreement from Greenphire.

Greenphire is a company working with Temple University to manage and process payments. Greenphire will be given your name, address, and date of birth. They will use this information only as part of the payment system, and it will not be given or sold to any other company. They will not receive any information about your health status or the study in which you are participating. This information will not be associated with the information or data you provide for this research. It will be stored separately from your data, it will not be linked in any way, and your identifying information will be destroyed within 1 year of study completion. If you would prefer not to provide this identifying information you may take part in this research if you agree to not be paid.

Federal tax law requires you to report this payment as income to the Internal Revenue Service. We are required to report payments more than $599.00, to the Internal Revenue Service and you will be sent a Form 1099-MISC if your total payment from Temple University is more than $599.00 for the year.

If you have questions about your rights as a research subject, or you have concerns or suggestions, and you want to talk to someone other than the researchers, you may contact the Temple University Institutional Review Board by phone at (215) 707-3390 or by email at irb@temple.edu.

Do you consent to participate in this survey?

◯ Yes
◯ No

Demographic Information

Please provide us with the following demographic information about yourself:

Age: Please enter your age in years.

____

Race/Ethnicity: How do you identify? Please select the choice(s) that best describe you. You may enter more than one answer.

- ☐ Black/African American
- ☐ Latinx
- ☐ White
- ☐ Multiracial
- ☐ Asian American and/or Pacific Islander
- ☐ Native American
- ☐ Prefer to self-describe or Other

If you prefer to self-describe your race/ethnicity, or your race/ethnicity best fits into another category, please specify your response here:

____

Gender: How do you identify?

- ◯ Female
- ◯ Non-binary
- ◯ Male
- ◯ Prefer to self-describe or Other

If you prefer to self-describe your gender, or your gender best fits another category, please specify your response here:

____

Relevant Expertise: Please share your expertise on the subject of media reporting on firearm violence (check all that apply):

- ☐ Lived experience expert (including firearm injury survivor and/or co-victim)
- ☐ Journalist (including reporter, photographer, editor, producer, community journalist, etc.)
- ☐ Scholar (including journalism and communications scholar, public health scholar, medicine scholar)
- ☐ Other

Please specify any relevant experience that falls into the other category:

____

We are interested in understanding specific elements of news reporting that may be potentially harmful to firearm-injured people, co-victims (including the loved-ones of firearm-injured people), communities impacted by firearm violence, and news audiences more generally.

Please answer the following questions using your expertise from personal lived experience, professional experience, and/or existing research and scholarship. There will be opportunities to share your thoughts in more depth as text entries throughout the survey. Please fill out these text entries, as your answers will provide us with important details that may not be captured in the questions that ask you to select answers from a list. You will also be asked to suggest additional harmful news elements that we may have overlooked.

For the following statements, please specify the extent to which you agree, disagree, or are neutral using the 7-point scale.

Graphic news content about firearm violence (such as a video of a shooting or a detailed description of a crime scene) could cause harm to:

	Strongly Agree	Agree	Somewhat Agree	Neither Agree nor Disagree	Somewhat Disagree	Disagree	Strongly Disagree
Firearm-injured people and/or co-victims involved in the shooting being reported on	○	○	○	○	○	○	○
Firearm-injured people and/or co-victims who have been affected by previous shootings	○	○	○	○	○	○	○
News audiences viewing, reading, and/or listening to the content and/or to society at large	○	○	○	○	○	○	○

Please share why you chose the answers you did for the previous 3 questions, including any research and/or personal experience that informs your perspective and the specific type of harm(s) this content may cause (if applicable). ______________________________

For the following statements, please specify the extent to which you agree, disagree, or are neutral using the 7-point scale.

News coverage of a shooting that includes information on the clinical condition of a firearm-injured person (e.g. "critical" or "stable") could cause harm to:

	Strongly Agree	Agree	Somewhat Agree	Neither Agree nor Disagree	Somewhat Disagree	Disagree	Strongly Disagree
Firearm-injured people and/or co-victims involved in the shooting being reported on	◯	◯	◯	◯	◯	◯	◯
Firearm-injured people and/or co-victims who have been affected by previous shootings	◯	◯	◯	◯	◯	◯	◯
News audiences viewing, reading and/or listening to the content and/or to society at large	◯	◯	◯	◯	◯	◯	◯

Please share why you chose the answers you did for the previous 3 questions, including any research and/or personal experience that informs your perspective and the specific type of harm(s) this content may cause (if applicable).

____

For the following statements, please specify the extent to which you agree, disagree, or are neutral using the 7-point scale.

News coverage of a shooting that includes information on the number of gunshot wounds of a firearm-injured person could cause harm to:

	Strongly Agree	Agree	Somewhat Agree	Neither Agree nor Disagree	Somewhat Disagree	Disagree	Strongly Disagree
Firearm-injured people and/or co-victims involved in the shooting being reported on	○	○	○	○	○	○	○
Firearm-injured people and/or co-victims who have been affected by previous shootings	○	○	○	○	○	○	○
News audiences viewing, reading and/or listening to the content and/or to society at large	○	○	○	○	○	○	○

Please share why you chose the answers you did for the previous 3 questions, including any research and/or personal experience that informs your perspective and the specific type of harm(s) this content may cause (if applicable). __________________________________

For the following statements, please specify the extent to which you agree, disagree, or are neutral using the 7-point scale.

News coverage of a shooting that includes information on the name of the treating hospital of a firearm-injured person could cause harm to:

	Strongly Agree	Agree	Somewhat Agree	Neither Agree nor Disagree	Somewhat Disagree	Disagree	Strongly Disagree
Firearm-injured people and/or co-victims involved in the shooting being reported on	○	○	○	○	○	○	○
Firearm-injured people and/or co-victims who have been affected by previous shootings	○	○	○	○	○	○	○
News audiences viewing, reading and/or listening to the content and/or to society at large	○	○	○	○	○	○	○

Please share why you chose the answers you did for the previous 3 questions, including any research and/or personal experience that informs your perspective and the specific type of harm(s) this content may cause (if applicable). __________________________________

For the following statements, please specify the extent to which you agree, disagree, or are neutral using the 7-point scale.

News coverage of a shooting that includes information on the relationship between the firearm-injured person and the shooter could cause harm to:

	Strongly Agree	Agree	Somewhat Agree	Neither Agree nor Disagree	Somewhat Disagree	Disagree	Strongly Disagree
Firearm-injured people and/or co-victims involved in the shooting being reported on	○	○	○	○	○	○	○
Firearm-injured people and/or co-victims who have been affected by previous shootings	○	○	○	○	○	○	○
News audiences viewing, reading and/or listening to the content and/or to society at large	○	○	○	○	○	○	○

Please share why you chose the answers you did for the previous 3 questions, including any research and/or personal experience that informs your perspective and the specific type of harm(s) this content may cause (if applicable). ______________________________

For the following statements, please specify the extent to which you agree, disagree, or are neutral using the 7-point scale.

News coverage of a shooting that includes a mugshot of the victim and/or perpetrator of a firearm-injured person could cause harm to:

	Strongly Agree	Agree	Somewhat Agree	Neither Agree nor Disagree	Somewhat Disagree	Disagree	Strongly Disagree
Firearm-injured people and/or co-victims involved in the shooting being reported on	○	○	○	○	○	○	○
Firearm-injured people and/or co-victims who have been affected by previous shootings	○	○	○	○	○	○	○
News audiences viewing, reading and/or listening to the content and/or to society at large	○	○	○	○	○	○	○

Please share why you chose the answers you did for the previous 3 questions, including any research and/or personal experience that informs your perspective and the specific type of harm(s) this content may cause (if applicable). ______________________________

For the following statements, please specify the extent to which you agree, disagree, or are neutral using the 7-point scale.

The absence of a follow-up story (e.g. an update on how a community has fared after a shooting or an interview with a survivor about their recovery) after initial "breaking news" coverage could cause harm to:

	Strongly Agree	Agree	Somewhat Agree	Neither Agree nor Disagree	Somewhat Disagree	Disagree	Strongly Disagree
Firearm-injured people and/or co-victims involved in the shooting being reported on	○	○	○	○	○	○	○
Firearm-injured people and/or co-victims who have been affected by previous shootings	○	○	○	○	○	○	○
News audiences viewing/reading or listening to the content and/or to society at large	○	○	○	○	○	○	○

Please share why you chose the answers you did for the previous 3 questions, including any research and/or personal experience that informs your perspective and the specific type of harm(s) this content may cause (if applicable).

____

For the following statements, please specify the extent to which you agree, disagree, or are neutral using the 7-point scale.

News coverage that focuses only on a specific shooting event and does NOT include context, root causes, or solutions to firearm violence (e.g. an episodic report) could cause harm to:

	Strongly Agree	Agree	Somewhat Agree	Neither Agree nor Disagree	Somewhat Disagree	Disagree	Strongly Disagree
Firearm-injured people and/or co-victims involved in the shooting being reported on	◯	◯	◯	◯	◯	◯	◯
Firearm-injured people and/or co-victims who have been affected by previous shootings	◯	◯	◯	◯	◯	◯	◯
News audiences viewing, reading and/or listening to the content and/or to society at large	◯	◯	◯	◯	◯	◯	◯

Please share why you chose the answers you did for the previous 3 questions, including any research and/or personal experience that informs your perspective and the specific type of harm(s) this content may cause (if applicable).

____

For the following statements, please specify the extent to which you agree, disagree, or are neutral using the 7-point scale.

News coverage of firearm violence that only or predominantly presents the perspectives of law enforcement representatives (e.g. police) could cause harm to:

	Strongly Agree	Agree	Somewhat Agree	Neither Agree nor Disagree	Somewhat Disagree	Disagree	Strongly Disagree
Firearm-injured people and/or co-victims involved in the shooting being reported on	○	○	○	○	○	○	○
Firearm-injured people and/or co-victims who have been affected by previous shootings	○	○	○	○	○	○	○
News audiences viewing, reading and/or listening to the content and/or to society at large	○	○	○	○	○	○	○

Please share why you chose the answers you did for the previous 3 questions, including any research and/or personal experience that informs your perspective and the specific type of harm(s) this content may cause (if applicable). __________________________________

For the following statements, please specify the extent to which you agree, disagree, or are neutral using the 7-point scale.

News coverage of a shooting that does NOT include the perspectives the injured person and/or their loved ones could cause harm to:

	Strongly Agree	Agree	Somewhat Agree	Neither Agree nor Disagree	Somewhat Disagree	Disagree	Strongly Disagree
Firearm-injured people and/or co-victims involved in the shooting being reported on	○	○	○	○	○	○	○
Firearm-injured people and/or co-victims who have been affected by previous shootings	○	○	○	○	○	○	○
News audiences viewing, reading and/or listening to the content and/or to society at large	○	○	○	○	○	○	○

Please share why you chose the answers you did for the previous 3 questions, including any research and/or personal experience that informs your perspective and the specific type of harm(s) this content may cause (if applicable). __________________________________

For the following statements, please specify the extent to which you agree, disagree, or are neutral using the 7-point scale.

News coverage of firearm violence that does NOT include the perspectives of people from the impacted community could cause harm to:

	Strongly Agree	Agree	Somewhat Agree	Neither Agree nor Disagree	Somewhat Disagree	Disagree	Strongly Disagree
Firearm-injured people and/or co-victims involved in the shooting being reported on	○	○	○	○	○	○	○
Firearm-injured people and/or co-victims who have been affected by previous shootings	○	○	○	○	○	○	○
News audiences viewing, reading and/or listening to the content and/or to society at large	○	○	○	○	○	○	○

Please share why you chose the answers you did for the previous 3 questions, including any research and/or personal experience that informs your perspective and the specific type of harm(s) this content may cause (if applicable).

____

For the following statements, please specify the extent to which you agree, disagree, or are neutral using the 7-point scale.

News coverage of firearm violence that does NOT explore potential solutions could cause harm to:

	Strongly Agree	Agree	Somewhat Agree	Neither Agree nor Disagree	Somewhat Disagree	Disagree	Strongly Disagree
Firearm-injured people and/or co-victims involved in the shooting being reported on	○	○	○	○	○	○	○
Firearm-injured people and/or co-victims who have been affected by previous shootings	○	○	○	○	○	○	○
News audiences viewing, reading and/or listening to the content and/or to society at large	○	○	○	○	○	○	○

Please share why you chose the answers you did for the previous 3 questions, including any research and/or personal experience that informs your perspective and the specific type of harm(s) this content may cause (if applicable).

____

We may not have included all of the harmful elements of firearm violence that are present in news reporting in this survey. Please use this space to list any other potentially harmful firearm violence news elements you have seen or know about. For each type of harmful content, please describe:

The type of harmful content The specific type of harm(s) this content may cause Who is being impacted by the harmful content You may click "Expand" to make the response box larger to fit your answers.

____

Please use this space to offer any information on harmful news elements on firearm violence that you have not included elsewhere.

____

Congratulations! You have completed the survey. Thank you so much for your input.

In order to compensate you for your time, we will need to collect some personal information from you, including your name, date of birth, and address. This information will be kept separate from your survey responses to protect your anonymity. The ClinCard for $75 will be mailed to you after you provide this information. Would you like to proceed?

○ Yes
○ No

Please click on the following link to provide your information for compensation:

Participant Information for Compensation

As a reminder, your personal information will be kept separate from your survey responses.

Once you have opened this link in a new browser tab, please CLICK SUBMIT BELOW to complete this survey.

Thank you for completing the survey. If you change your mind and would like to receive compensation for your participation, please contact Dr. Jessica Beard at jessica.beard@tuhs.temple.edu.

Please CLICK SUBMIT below when you are done.
