# Supplementary material for: Defining harmful news reporting on community firearm violence: A modified Delphi consensus study
Source: PLoS One. 2024 Dec 18;19(12):e0316026. doi: 10.1371/journal.pone.0316026 (PMC11654925; doi:10.1371/journal.pone.0316026)
Supplement: S2 Appendix — (PDF) [file pone.0316026.s002.pdf]

Round 2 Delphi

Welcome Delphi Panelists!

As a reminder, this is a Temple University research study conducted by Jessica Beard, MD, MPH and her research team. Your participation in this research study is voluntary. The purpose of this study is to understand the perspectives of experts like you on what constitutes harmful news reporting on firearm violence in Philadelphia. The group of experts in this study is called a Delphi Panel. You have been invited to participate as an expert in this Delphi Panel because you are one or more of the following: a survivor or co-victim with lived experience of firearm violence, a journalist, and/or an academic expert in this area.

This study include three rounds of surveys. You are being invited to complete the second round survey. This survey will take approximately 30 minutes to complete. No personally identifiable information will be recorded about you, and the investigators will not attempt to re-identify any of your information. However, there is a possibility that researchers could determine your identity based on the demographic information you provide because the participant pool is relatively small. This means there is a small risk of loss of your confidentiality. Your de-identified data will be kept for use in future research and shared with other researchers. Your responses to the survey will be combined with those of the other panelists and summarized in a report to further protect your anonymity.

Upon completion of this survey, you will receive $75 to compensate you for your time. Payments will be made to you using ClinCard, a secure, reloadable MasterCard debit card supported by Greenphire. You may use this card online or at any store that accepts MasterCard. We will mail you the card. You will be given one card for the entire time of your participation, which may be used again if you participate in the subsequent surveys. Be sure to read the information included with your ClinCard, including the cardholder agreement from Greenphire.

Greenphire is a company working with Temple University to manage and process payments. Greenphire will be given your name, address, and date of birth. They will use this information only as part of the payment system, and it will not be given or sold to any other company. They will not receive any information about your health status or the study in which you are participating. This information will not be associated with the information or data you provide for this research. It will be stored separately from your data, it will not be linked in any way, and your identifying information will be destroyed within 1 year of study completion. If you would prefer not to provide this identifying information you may take part in this research if you agree to not be paid.

Federal tax law requires you to report this payment as income to the Internal Revenue Service. We are required to report payments more than $599.00, to the Internal Revenue Service and you will be sent a Form 1099-MISC if your total payment from Temple University is more than $599.00 for the year.

If you have questions about your rights as a research subject, or you have concerns or suggestions, and you want to talk to someone other than the researchers, you may contact the Temple University Institutional Review Board by phone at (215) 707-3390 or by email at irb@temple.edu.

Do you agree to participate in this survey?

◯ Yes
◯ No

Demographic Information

Please provide us with the following demographic information about yourself:

Age: Please enter your age in years.

____

Race/Ethnicity: How do you identify? Please select the choice(s) that best describe you. You may enter more than one answer.

- ☐ Black/African American
- ☐ Latinx
- ☐ White
- ☐ Multiracial
- ☐ Asian American and/or Pacific Islander
- ☐ Native American
- ☐ Prefer to self-describe or Other

If you prefer to self-describe your race/ethnicity, or your race/ethnicity best fits into another category, please specify your response here:

____

Gender: How do you identify?

- ◯ Female
- ◯ Non-binary
- ◯ Male
- ◯ Prefer to self-describe or Other

If you prefer to self-describe your gender, or your gender best fits another category, please specify your response here:

____

Relevant Expertise: Please share your expertise on the subject of media reporting on firearm violence (check all that apply):

- ☐ Lived experience expert (including firearm injury survivor and/or co-victim)
- ☐ Journalist (including reporter, photographer, editor, producer, community journalist, etc.)
- ☐ Scholar (including journalism and communications scholar, public health scholar, medicine scholar)
- ☐ Other

Please specify any relevant experience that falls into the other category:

____

Thank you for your responses to our Round 1 Survey.

In Round 2, we are interested in learning about how you view the SEVERITY of each harmful reporting element and how that severity COMPARES across the reporting elements we looked at in Round 1.

As a reminder, the THREE levels of harm we are considering in this survey are harm to:

(1) Firearm-injured people and/or co-victims (including the loved-ones of firearm injured people) involved in the shooting being reported on;

(2) Firearm-injured people and/or co-victims who have been affected by previous shootings;

(3) News audiences viewing/reading or listening to the content and/or to society at large;

Please answer the following questions using your expertise from personal lived experience, professional experience, and/or existing research and scholarship.

Based on your feedback, we have added questions on your views of harmful visual imagery towards the end of the survey. You will also have the opportunity to contribute your perspectives through open-ended questions throughout the survey.

The next THREE questions focus on the impact of harmful reporting on firearm injured people and/or co-victims involved in the shooting being reported on.

Please consider this population when answering the questions.

On a scale of 0 to 10, please rate the following news content elements on their potential to harm firearm-injured people and/or co-victims involved in the shooting being reported on.

A score of 0 indicates no harm and 10 indicates extreme harm.

	No Harm 0	1	Slight Harm 2	3	4	Some Harm 5	6	7	Significant Harm 8	9	Extreme Harm 10
Coverage does NOT include the perspectives of the firearm-injured person and/or their loved ones	◯	◯	◯	◯	◯	◯	◯	◯	◯	◯	◯
Coverage does NOT include the perspectives of people from the impacted community	◯	◯	◯	◯	◯	◯	◯	◯	◯	◯	◯
Coverage does NOT include a follow-up story after the initial "breaking news" coverage	◯	◯	◯	◯	◯	◯	◯	◯	◯	◯	◯
Coverage does NOT explore potential solutions to firearm violence	◯	◯	◯	◯	◯	◯	◯	◯	◯	◯	◯
Coverage focuses on a specific shooting event and does not include context, root causes, or solutions to firearm violence (episodic report)	◯	◯	◯	◯	◯	◯	◯	◯	◯	◯	◯
Coverage includes information on the clinical condition of a firearm-injured person ("critical" or "stable")	◯	◯	◯	◯	◯	◯	◯	◯	◯	◯	◯
Coverage includes the number of gunshot wounds of a firearm-injured person	◯	◯	◯	◯	◯	◯	◯	◯	◯	◯	◯
Coverage includes the name of the treating hospital	◯	◯	◯	◯	◯	◯	◯	◯	◯	◯	◯
Coverage includes relationship between the firearm-injured person and the perpetrator	◯	◯	◯	◯	◯	◯	◯	◯	◯	◯	◯
Coverage only or predominantly includes the perspectives of law enforcement and/or police	◯	◯	◯	◯	◯	◯	◯	◯	◯	◯	◯

Coverage includes graphic and/or explicit news content about firearm violence (video of a shooting, still photo of a body, audio of screaming)	◯	◯	◯	◯	◯	◯	◯	◯	◯	◯	◯
Coverage includes a mugshot of the suspected perpetrator of firearm violence	◯	◯	◯	◯	◯	◯	◯	◯	◯	◯	◯

Think about the news content elements that are the MOST HARMFUL to firearm-injured people and/or co-victims involved in the shooting being reported on.

Select the top three MOST HARMFUL news elements from your perspective (based on personal experience, professional experience and/or research). Please check three boxes below.

- ☐ Coverage does NOT include the perspectives of the firearm-injured person and/or their loved ones
- ☐ Coverage does NOT include the perspectives of people from the impacted community
- ☐ Coverage does NOT include a follow-up story after the initial "breaking news" coverage
- ☐ Coverage does NOT explore potential solutions to firearm violence
- ☐ Coverage focuses on a specific shooting event and does not include context, root causes, or solutions to firearm violence (episodic report)
- ☐ Coverage includes information on the clinical condition of a firearm-injured person ("critical" or "stable")
- ☐ Coverage includes the number of gunshot wounds of a firearm-injured person
- ☐ Coverage includes the name of the treating hospital
- ☐ Coverage includes relationship between the firearm-injured person and the perpetrator
- ☐ Coverage only or predominantly includes the perspectives of law enforcement and/or police
- ☐ Coverage includes graphic and/or explicit news content about firearm violence (video of a shooting, still photo of a body, audio of screaming)
- ☐ Coverage includes a mugshot of the suspected perpetrator of firearm violence

---

Please share why you chose the answers you did for the previous three questions, including any research, personal and/or professional experience that informs your perspective on why certain news content elements are more or less harmful for firearm-injured people and/or co-victims invovled in the shooting being reported on.

____

The next THREE questions focus on the impact of harmful reporting on firearm-injured people and/or co-victims who have been affected by previous shootings.

Please consider this population when answering the questions.

On a scale of 0 to 10, please rate the following news content elements on their potential to harm firearm-injured people and/or co-victims who have been affected by previous shootings.

A score of 0 indicates no harm and 10 indicates extreme harm.

	No Harm 0	1	Slight Harm 2	3	4	Some Harm 5	6	7	Significant Harm 8	9	Extreme Harm 10
Coverage does NOT include the perspectives of the firearm-injured person and/or their loved ones											
Coverage does NOT include the perspectives of people from the impacted community											
Coverage does NOT include a follow-up story after the initial "breaking news" coverage											
Coverage does NOT explore potential solutions to firearm violence											
Coverage focuses on a specific shooting event and does not include context, root causes, or solutions to firearm violence (episodic report)											
Coverage includes information on the clinical condition of a firearm-injured person ("critical" or "stable")											
Coverage includes the number of gunshot wounds of a firearm-injured person											
Coverage includes the name of the treating hospital											
Coverage includes relationship between the firearm-injured person and the perpetrator											
Coverage only or predominantly includes the perspectives of law enforcement and/or police											
Coverage includes graphic and/or explicit news content about firearm violence (video of a shooting, still photo of a body, audio of screaming)											
Coverage includes a mugshot of the suspected perpetrator of firearm violence											

Think about the news content elements that are the MOST HARMFUL to firearm-injured people and/or co-victims who have been affected by previous shootings.

Select the top three MOST HARMFUL news elements from your perspective (based on personal experience, professional experience and/or research). Please check three boxes below.

- ☐ Coverage does NOT include the perspectives of the firearm-injured person and/or their loved ones
- ☐ Coverage does NOT include the perspectives of people from the impacted community
- ☐ Coverage does NOT include a follow-up story after the initial "breaking news" coverage
- ☐ Coverage does NOT explore potential solutions to firearm violence
- ☐ Coverage focuses on a specific shooting event and does not include context, root causes, or solutions to firearm violence (episodic report)
- ☐ Coverage includes information on the clinical condition of a firearm-injured person ("critical" or "stable")
- ☐ Coverage includes the number of gunshot wounds of a firearm-injured person
- ☐ Coverage includes the name of the treating hospital
- ☐ Coverage includes relationship between the firearm-injured person and the perpetrator
- ☐ Coverage only or predominantly includes the perspectives of law enforcement and/or police
- ☐ Coverage includes graphic and/or explicit news content about firearm violence (video of a shooting, still photo of a body, audio of screaming)
- ☐ Coverage includes a mugshot of the suspected perpetrator of firearm violence

Please share why you chose the answers you did for the previous three questions, including any research, personal and/or professional experience that informs your perspective on why certain news content elements are more or less harmful for firearm-injured people and/or co-victims who have been affected by previous shootings. ____

The following THREE questions focus on the impact of harmful reporting on news audiences viewing/reading or listening to the content and/or to society at large.

Please consider this population when answering the questions.

On a scale of 0 to 10, please rate the following news content elements on their potential to harm news audiences viewing/reading or listening to the content and/or to society at large.

A score of 0 indicates no harm and 10 indicates extreme harm.

	No Harm 0	1	Slight Harm 2	3	4	Some Harm 5	6	7	Significant Harm 8	9	Extreme Harm 10
Coverage does NOT include the perspectives of the firearm-injured person and/or their loved ones	○	○	○	○	○	○	○	○	○	○	○
Coverage does NOT include the perspectives of people from the impacted community	○	○	○	○	○	○	○	○	○	○	○

Coverage does NOT include a follow-up story after the initial "breaking news" coverage	○	○	○	○	○	○	○	○	○	○	○
Coverage does NOT explore potential solutions to firearm violence	○	○	○	○	○	○	○	○	○	○	○
Coverage focuses on a specific shooting event and does not include context, root causes, or solutions to firearm violence (episodic report)	○	○	○	○	○	○	○	○	○	○	○
Coverage includes information on the clinical condition of a firearm-injured person ("critical" or "stable")	○	○	○	○	○	○	○	○	○	○	○
Coverage includes the number of gunshot wounds of a firearm-injured person	○	○	○	○	○	○	○	○	○	○	○
Coverage includes the name of the treating hospital	○	○	○	○	○	○	○	○	○	○	○
Coverage includes relationship between the firearm-injured person and the perpetrator	○	○	○	○	○	○	○	○	○	○	○
Coverage only or predominantly includes the perspectives of law enforcement and/or police	○	○	○	○	○	○	○	○	○	○	○
Coverage includes graphic and/or explicit news content about firearm violence (video of a shooting, still photo of a body, audio of screaming)	○	○	○	○	○	○	○	○	○	○	○
Coverage includes a mugshot of the suspected perpetrator of firearm violence	○	○	○	○	○	○	○	○	○	○	○

Think about the news content elements that are the MOST HARMFUL for news audiences viewing/reading or listening to the content and/or to society at large.

Select the top three MOST HARMFUL news elements from your perspective (based on personal experience, professional experience and/or research). Please check three boxes below.

☐ Coverage does NOT include the perspectives of the firearm-injured person and/or their loved ones
☐ Coverage does NOT include the perspectives of people from the impacted community
☐ Coverage does NOT include a follow-up story after the initial "breaking news" coverage
☐ Coverage does NOT explore potential solutions to firearm violence
☐ Coverage focuses on a specific shooting event and does not include context, root causes, or solutions to firearm violence (episodic report)
☐ Coverage includes information on the clinical condition of a firearm-injured person ("critical" or "stable")
☐ Coverage includes the number of gunshot wounds of a firearm-injured person
☐ Coverage includes the name of the treating hospital
☐ Coverage includes relationship between the firearm-injured person and the perpetrator
☐ Coverage only or predominantly includes the perspectives of law enforcement and/or police
☐ Coverage includes graphic and/or explicit news content about firearm violence (video of a shooting, still photo of a body, audio of screaming)
☐ Coverage includes a mugshot of the perpetrator of firearm violence

---

Please share why you chose the answers you did for the previous three questions, including any research, personal and/or professional experience that informs your perspective on why certain news content elements are more or less harmful for news audiences viewing/reading or listening to the content and/or to society at large.

____

In the Round 1 Questionnaire, most respondents found graphic content to be harmful. We are interested in learning more about your perspectives on what constitutes graphic and/or explicit news content about firearm violence.

Which of the following would you consider to be harmful graphic and/or explicit content? Content in this question includes any video, still photographs, audio from scene, and audio narration. Please check all that apply.

☐ The actual shooting incident as it unfolds
☐ The crime scene (without any people)
☐ Blood at the crime scene or on objects
☐ Police investigating the crime scene or in a neighborhood
☐ General footage of police and related items (flashing lights, crime scene tape, handcuffs)
☐ An injured survivor
☐ Someone being loaded into or out of an emergency transport vehicle (e.g. ambulance, police car)
☐ The covered body of a deceased victim(s)
☐ The uncovered body of a deceased victim(s)
☐ Family or friends crying about a firearm-injured person
☐ A memorial at the site of a shooting
☐ A funeral or memorial service

Please describe any other news content you consider to be graphic, explicit, and harmful for firearm injured people, co-victims, community members, news audiences, and/or society.

____

We are also interested in learning whether you think graphic and/or explicit news content is more or less harmful when communicated using certain formats (e.g. audio, video).

On a scale of 0 to 10, please rate the following news content elements on their potential to harm firearm-injured people and/or co-victims involved in the shooting being reported on.

A score of 0 indicates no harm and 10 indicates extreme harm.

	No Harm 0	1	Slight Harm 2	3	4	Some Harm 5	6	7	Significant Harm 8	9	Extreme Harm 10
Coverage includes disturbing video of firearm violence	○	○	○	○	○	○	○	○	○	○	○
Coverage includes disturbing still photographs of firearm violence	○	○	○	○	○	○	○	○	○	○	○
Coverage includes disturbing audio of firearm violence	○	○	○	○	○	○	○	○	○	○	○
Coverage includes a disturbing detailed verbal description of firearm violence	○	○	○	○	○	○	○	○	○	○	○

On a scale of 0 to 10, please rate the following news content elements on their potential to harm firearm-injured people and/or co-victims who have been affected by previous shootings.

A score of 0 indicates no harm and 10 indicates extreme harm.

	No Harm 0	1	Slight Harm 2	3	4	Some Harm 5	6	7	Significant Harm 8	9	Extreme Harm 10
Coverage includes disturbing video of firearm violence	○	○	○	○	○	○	○	○	○	○	○
Coverage includes disturbing still photographs of firearm violence	○	○	○	○	○	○	○	○	○	○	○
Coverage includes disturbing audio of firearm violence	○	○	○	○	○	○	○	○	○	○	○
Coverage includes a disturbing detailed verbal description of firearm violence	○	○	○	○	○	○	○	○	○	○	○

On a scale of 0 to 10, please rate the following news content elements on their potential to harm news audiences viewing/reading or listening to the content and/or to society at large.

A score of 0 indicates no harm and 10 indicates extreme harm.

	No Harm 0	1	Slight Harm 2	3	4	Some Harm 5	6	7	Significant Harm 8	9	Extreme Harm 10
Coverage includes disturbing video of firearm violence	○	○	○	○	○	○	○	○	○	○	○
Coverage includes disturbing still photographs of firearm violence	○	○	○	○	○	○	○	○	○	○	○
Coverage includes disturbing audio of firearm violence	○	○	○	○	○	○	○	○	○	○	○

Coverage includes a disturbing detailed verbal description of firearm violence	○	○	○	○	○	○	○	○	○	○	○

Please use this space to add any comments you may have about harmful content, severity of harm, and levels of harms that we did not cover in this survey.

You may click "Expand" to make the response box larger to fit your answers.

____

Please use this space to offer any information on harmful news elements on firearm violence that you have not included elsewhere.

____

Congratulations! You have completed the survey. Thank you so much for your input.

In order to compensate you for your time, we will need to collect some personal information from you, including your name, date of birth, and address. This information will be kept separate from your survey responses to protect your anonymity. The ClinCard for $75 will be mailed to you after you provide this information. Would you like to proceed?

○ Yes
○ No

Please click on the following link to provide your information for compensation:

Participant Information for Compensation

As a reminder, your personal information will be kept separate from your survey responses.

Once you have opened this link in a new browser tab, please CLICK SUBMIT BELOW to complete this survey.

Thank you for completing the survey. If you change your mind and would like to receive compensation for your participation, please contact Dr. Jessica Beard at jessica.beard@tuhs.temple.edu.

Please CLICK SUBMIT below when you are done.
