# Supplementary material for: Defining harmful news reporting on community firearm violence: A modified Delphi consensus study
Source: PLoS One. 2024 Dec 18;19(12):e0316026. doi: 10.1371/journal.pone.0316026 (PMC11654925; doi:10.1371/journal.pone.0316026)
Supplement: S3 Appendix — (PDF) [file pone.0316026.s003.pdf]

Round 3 Delphi

Welcome Delphi Panelists!

As a reminder, this is a Temple University research study conducted by Jessica Beard, MD, MPH and her research team. Your participation in this research study is voluntary. The purpose of this study is to understand the perspectives of experts like you on what constitutes harmful news reporting on firearm violence in Philadelphia. The group of experts in this study is called a Delphi Panel. You have been invited to participate as an expert in this Delphi Panel because you are one or more of the following: a survivor or co-victim with lived experience of firearm violence, a journalist, and/or an academic expert in this area.

This study include three rounds of surveys. You are being invited to complete the third round survey. This survey will take approximately 30 minutes to complete. No personally identifiable information will be recorded about you, and the investigators will not attempt to re-identify any of your information. However, there is a possibility that researchers could determine your identity based on the demographic information you provide because the participant pool is relatively small. This means there is a small risk of loss of your confidentiality. Your de-identified data will be kept for use in future research and shared with other researchers. Your responses to the survey will be combined with those of the other panelists and summarized in a report to further protect your anonymity.

Upon completion of this survey, you will receive $75 to compensate you for your time. Payments will be made to you using ClinCard, a secure, reloadable MasterCard debit card supported by Greenphire. You may use this card online or at any store that accepts MasterCard. We will reload your card or mail you a replacement card if needed. Be sure to read the information included with your ClinCard, including the cardholder agreement from Greenphire.

Greenphire is a company working with Temple University to manage and process payments. Greenphire will be given your name, address, and date of birth. They will use this information only as part of the payment system, and it will not be given or sold to any other company. They will not receive any information about your health status or the study in which you are participating. This information will not be associated with the information or data you provide for this research. It will be stored separately from your data, it will not be linked in any way, and your identifying information will be destroyed within 1 year of study completion. If you would prefer not to provide this identifying information you may take part in this research if you agree to not be paid.

Federal tax law requires you to report this payment as income to the Internal Revenue Service. We are required to report payments more than $599.00, to the Internal Revenue Service and you will be sent a Form 1099-MISC if your total payment from Temple University is more than $599.00 for the year.

If you have questions about your rights as a research subject, or you have concerns or suggestions, and you want to talk to someone other than the researchers, you may contact the Temple University Institutional Review Board by phone at (215) 707-3390 or by email at irb@temple.edu.

Do you agree to participate in this survey?

- ◯ Yes
- ◯ No

Demographic Information

Please provide us with the following demographic information about yourself:

Age: Please enter your age in years.	__________________________
Race/Ethnicity: How do you identify? Please select the choice(s) that best describe you. You may enter more than one answer.	☐ Black/African American<br>☐ Latinx<br>☐ White<br>☐ Multiracial<br>☐ Asian American and/or Pacific Islander<br>☐ Native American<br>☐ Prefer to self-describe or Other
If you prefer to self-describe your race/ethnicity, or your race/ethnicity best fits into another category, please specify your response here:	__________________________
Gender: How do you identify?	◯ Female<br>◯ Non-binary<br>◯ Male<br>◯ Prefer to self-describe or Other
If you prefer to self-describe your gender, or your gender best fits another category, please specify your response here:	__________________________
Relevant Expertise: Please share your expertise on the subject of media reporting on firearm violence (check all that apply):	☐ Lived experience expert (including firearm injury survivor and/or co-victim)<br>☐ Journalist (including reporter, photographer, editor, producer, community journalist, etc.)<br>☐ Scholar (including journalism and communications scholar, public health scholar, medicine scholar)<br>☐ Other
Please specify any relevant experience that falls into the other category:	________________________________

Thank you for your responses to our Round 2 Survey.

In Round 3, we are interested in achieving consensus (agreement) among participants in this Delphi Panel on what constitutes harmful reporting on firearm violence. Specifically, we are looking for agreement among Delphi panelists regarding the SEVERITY of each harmful reporting element for each level of harm.

As a reminder, the THREE levels of harm we are considering in this survey are harm to:

(1) Firearm-injured people and/or co-victims (including the loved-ones of firearm injured people) involved in the shooting being reported on;

(2) Firearm-injured people and/or co-victims who have been affected by previous shootings;

(3) News audiences viewing/reading or listening to the content and/or to society at large;

Please answer the following questions using your expertise from personal lived experience, professional experience, and/or existing research and scholarship.

If you do not agree with the proposed severity rating across levels of harm, you will be given the opportunity to explain why you are outside consensus in open ended questions.

In the Round 2 Survey, you rated each harmful content element on a scale of 0 to 10. A score of 0 meant no harm and 10 meant extreme harm.

For each type of harmful content and level of harm, we calculated the median severity rating according to you and the other 20 participants in this Delphi Panel. A median is the middle number in a set of data that is ordered from least to greatest and best describes how this panel as a whole views the severity of each harmful element.

We have assigned severity ratings of harm for type of news content and harm level as follows:

Rating of 8 to 10 represents SEVERE HARM Rating of 5 to 7 represents MODERATE HARM Rating of less than 5 represents MILD HARM The table below summarizes the findings from the Delphi Panel, including your responses. In this table, you can see the serverity rating for each harmful news content element by level according to the Delphi panelists.

FIP is Firearm-injured person

[Attachment: "Round 2 Table.pdf"]

The following three questions concern potential harm caused by news coverage that does NOT include the perspectives of the firearm-injured person and/or their loved ones.

Please only select disagree if you strongly believe that the average rating of the Delphi panel is incorrect.

The Delphi panelists found that coverage that does NOT include the perspectives of the firearm-injured person and/or their loved ones has the potential to cause SEVERE HARM to firearm-injured people and/or co-victims involved in the shooting being reported on.

Please specify if you agree with the Delphi panel's rating of SEVERE HARM.

◯ Agree
◯ Disagree

Please describe your reasoning for being outside the consensus here.

____

The Delphi panelists found that coverage that does NOT include the perspectives of the firearm-injured person and/or their loved ones has the potential to cause MODERATE HARM to firearm-injured people and/or co-victims who have been affected by previous shootings.

Please specify if you agree with the Delphi panel's rating of MODERATE HARM.

◯ Agree
◯ Disagree

Please describe your reasoning for being outside the consensus here.

____

The Delphi panelists found that coverage that does NOT include the perspectives of the firearm-injured person and/or their loved ones has the potential to cause: MODERATE HARM to news audiences viewing/reading or listening to the content and/or society at large.

Please specify if you agree with the Delphi panel's rating of MODERATE HARM.

◯ Agree
◯ Disagree

Please describe your reasoning for being outside the consensus here.

____

The following three questions concern potential harm caused by news coverage that does NOT include the perspectives of people from the impacted community.

Please only select disagree if you strongly believe that the average rating of the Delphi panel is incorrect.

The Delphi panelists found that coverage that does NOT include the perspectives of people from the impacted community has the potential to cause SEVERE HARM to firearm-injured people and/or co-victims involved in the shooting being reported on.

Please specify if you agree with the Delphi panel's rating of SEVERE HARM.

◯ Agree
◯ Disagree

Please describe your reasoning for being outside the consensus here.

____

The Delphi panelists found that coverage that does NOT include the perspectives of people from the impacted community has the potential to cause MODERATE HARM to firearm-injured people and/or co-victims who have been affected by previous shootings.

Please specify if you agree with the Delphi panel's rating of MODERATE HARM.

◯ Agree
◯ Disagree

Please describe your reasoning for being outside the consensus here.

____

The Delphi panelists found that coverage that does NOT include the perspectives of people from the impacted community has the potential to cause MODERATE HARM to news audiences viewing/reading or listening to the content and/or society at large.

Please specify if you agree with the Delphi panel's rating of MODERATE HARM.

◯ Agree
◯ Disagree

Please describe your reasoning for being outside the consensus here.

____

The following three questions concern potential harm caused by news coverage that does NOT include a follow-up story after the initial "breaking news" coverage.

Please only select disagree if you strongly believe that the average rating of the Delphi panel is incorrect.

The Delphi panelists found that coverage that does NOT include a follow-up story after the initial "breaking news" coverage has the potential to cause SEVERE HARM to firearm-injured people and/or co-victims involved in the shooting being reported on.

Please specify if you agree with the Delphi panel's rating of SEVERE HARM.

◯ Agree
◯ Disagree

Please describe your reasoning for being outside the consensus here. ______________________________________

The Delphi panelists found that coverage that does NOT include a follow-up story after the initial "breaking news" coverage has the potential to cause MODERATE HARM to firearm-injured people and/or co-victims who have been affected by previous shootings.

Please specify if you agree with the Delphi panel's rating of MODERATE HARM.

◯ Agree
◯ Disagree

Please describe your reasoning for being outside the consensus here. ______________________________________

The Delphi panelists found that coverage that does NOT include a follow-up story after the initial "breaking news" coverage has the potential to cause MODERATE HARM to news audiences viewing/reading or listening to the content and/or society at large.

Please specify if you agree with the Delphi panel's rating of MODERATE HARM.

◯ Agree
◯ Disagree

Please describe your reasoning for being outside the consensus here. ______________________________________

The following three questions concern potential harm caused by news coverage that does NOT explore potential solutions to firearm violence.

Please only select disagree if you strongly believe that the average rating of the Delphi panel is incorrect.

The Delphi panelists found that coverage that does NOT explore potential solutions to firearm violence has the potential to cause SEVERE HARM to firearm-injured people and/or co-victims involved in the shooting being reported on.

Please specify if you agree with the Delphi panel's rating of SEVERE HARM.

◯ Agree
◯ Disagree

Please describe your reasoning for being outside the consensus here. ______________________________

The Delphi panelists found that coverage that does NOT explore potential solutions to firearm violence has the potential to cause SEVERE HARM to firearm-injured people and/or co-victims who have been affected by previous shootings.

Please specify if you agree with the Delphi panel's rating of SEVERE HARM.

◯ Agree
◯ Disagree

Please describe your reasoning for being outside the consensus here. ______________________________

The Delphi panelists found that coverage that does NOT explore potential solutions to firearm violence has the potential to cause SEVERE HARM to news audiences viewing/reading or listening to the content and/or society at large.

Please specify if you agree with the Delphi panel's rating of SEVERE HARM.

◯ Agree
◯ Disagree

Please describe your reasoning for being outside the consensus here. ______________________________

The following three questions concern potential harm caused by news coverage that focuses on a specific shooting event and does not include context, root causes, or solutions for firearm violence (episodic report).

Please only select disagree if you strongly believe that the average rating of the Delphi panel is incorrect.

The Delphi panelists found that coverage that focuses on a specific shooting event and does not include context, root causes, or solutions for firearm violence (episodic report) has the potential to cause SEVERE HARM to firearm-injured people and/or co-victims involved in the shooting being reported on.

Please specify if you agree with the Delphi panel's rating of SEVERE HARM.

◯ Agree
◯ Disagree

Please describe your reasoning for being outside the consensus here.

____

The Delphi panelists found that coverage that focuses on a specific shooting event and does not include context, root causes, or solutions for firearm violence (episodic report) has the potential to cause SEVERE HARM (median severity score 8 out of 10) to firearm-injured people and/or co-victims who have been affected by previous shootings.

Please specify if you agree with the Delphi panel's rating of SEVERE HARM.

◯ Agree
◯ Disagree

Please describe your reasoning for being outside the consensus here.

____

The Delphi panelists found that coverage that focuses on a specific shooting event and does not include context, root causes, or solutions for firearm violence (episodic report) has the potential to cause SEVERE HARM to news audiences viewing/reading or listening to the content and/or society at large.

Please specify if you agree with the Delphi panel's rating of SEVERE HARM.

◯ Agree
◯ Disagree

Please describe your reasoning for being outside the consensus here.

____

The following three questions concern potential harm caused by news coverage that includes information on the clinical condition of a firearm-injured person (e.g. "critical" or "stable").

Please only select disagree if you strongly believe that the average rating of the Delphi panel is incorrect.

The Delphi panelists found that coverage that includes clinical information about the firerm-injured person, including clinical condition (e.g. "critical" or "stable") has the potential to cause MODERATE HARM to firearm-injured people and/or co-victims involved in the shooting being reported on.

Please specify if you agree with the Delphi panel's rating of MODERATE HARM.

○ Agree
○ Disagree

Please describe your reasoning for being outside the consensus here.

____

The Delphi panelists found that coverage that includes clinical information about the firerm-injured person, including clinical condition (e.g. "critical" or "stable") has the potential to cause MODERATE HARM to firearm-injured people and/or co-victims who have been affected by previous shootings.

Please specify if you agree with the Delphi panel's rating of MODERATE HARM.

○ Agree
○ Disagree

Please describe your reasoning for being outside the consensus here.

____

The Delphi panelists found that coverage that includes clinical information about the firerm-injured person, including clinical condition (e.g. "critical" or "stable") has the potential to cause MILD HARM to news audiences viewing/reading or listening to the content and/or society at large.

Please specify if you agree with the Delphi panel's rating of MILD HARM.

○ Agree
○ Disagree

Please describe your reasoning for being outside the consensus here.

____

The following three questions concern potential harm caused by news coverage that includes information about the number of gunshot wounds of a firearm-injured person.

Please only select disagree if you strongly believe that the average rating of the Delphi panel is incorrect.

The Delphi panelists found that coverage that includes information about the number of gunshot wounds of a firearm-injured person has the potential to cause MODERATE HARM to firearm-injured people and/or co-victims involved in the shooting being reported on.

Please specify if you agree with the Delphi panel's rating of MODERATE HARM.

◯ Agree
◯ Disagree

Please describe your reasoning for being outside the consensus here.

____

The Delphi panelists found that coverage that includes information about the number of gunshot wounds of a firearm-injured person has the potential to cause MODERATE HARM to firearm-injured people and/or co-victims who have been affected by previous shootings.

Please specify if you agree with the Delphi panel's rating of MODERATE HARM.

◯ Agree
◯ Disagree

Please describe your reasoning for being outside the consensus here.

____

The Delphi panelists found that coverage that includes the number of gunshot wounds of a firearm-injured person has the potential to cause MODERATE HARM to news audiences viewing/reading or listening to the content and/or society at large.

Please specify if you agree with the Delphi panel's rating of MODERATE HARM.

◯ Agree
◯ Disagree

Please describe your reasoning for being outside the consensus here.

____

The following three questions concern potential harm caused by news coverage that includes the name of the treating hospital of a firearm-injured person.

Please only select disagree if you strongly believe that the average rating of the Delphi panel is incorrect.

The Delphi panelists found that coverage that includes the name of the treating hospital of a firearm-injured person has the potential to cause SEVERE HARM to firearm-injured people and/or co-victims involved in the shooting being reported on.

Please specify if you agree with the Delphi panel's rating of SEVERE HARM.

◯ Agree
◯ Disagree

Please describe your reasoning for being outside the consensus here.

____

The Delphi panelists found that coverage that includes the name of the treating hospital of a firearm-injured person has the potential to cause MODERATE HARM to firearm-injured people and/or co-victims who have been affected by previous shootings.

Please specify if you agree with the Delphi panel's rating of MODERATE HARM.

◯ Agree
◯ Disagree

Please describe your reasoning for being outside the consensus here.

____

The Delphi panelists found that coverage that includes the name of the treating hospital of a firearm-injured person has the potential to cause MILD HARM to news audiences viewing/reading or listening to the content and/or society at large.

Please specify if you agree with the Delphi panel's rating of MILD HARM.

◯ Agree
◯ Disagree

Please describe your reasoning for being outside the consensus here.

____

The following three questions concern potential harm caused by news coverage that includes information about the relationship between the firearm-injured person and the perpetrator.

Please only select disagree if you strongly believe that the average rating of the Delphi panel is incorrect.

The Delphi panelists found that coverage that includes information about the relationship between the firearm-injured person and the perpetrator has the potential to cause MODERATE HARM to firearm-injured people and/or co-victims involved in the shooting being reported on.

Please specify if you agree with the Delphi panel's rating of MODERATE HARM.

◯ Agree
◯ Disagree

Please describe your reasoning for being outside the consensus here.

____

The Delphi panelists found that coverage that includes information about the relationship between the firearm-injured person and the perpetrator has the potential to cause MODERATE HARM to firearm-injured people and/or co-victims who have been affected by previous shootings.

Please specify if you agree with the Delphi panel's rating of MODERATE HARM.

◯ Agree
◯ Disagree

Please describe your reasoning for being outside the consensus here.

____

The Delphi panelists found that coverage that includes information about the relationship between the firearm-injured person and the perpetrator has the potential to cause MODERATE HARM to news audiences viewing/reading or listening to the content and/or society at large.

Please specify if you agree with the Delphi panel's rating of MODERATE HARM.

◯ Agree
◯ Disagree

Please describe your reasoning for being outside the consensus here.

____

The following three questions concern potential harm caused by news coverage that only or predominantly includes the perspectives of law enforcement

Please only select disagree if you strongly believe that the average rating of the Delphi panel is incorrect.

The Delphi panelists found that coverage that only or predominantly includes the perspectives of law enforcement has the potential to cause SEVERE HARM to firearm-injured people and/or co-victims involved in the shooting being reported on.

Please specify if you agree with the Delphi panel's rating of SEVERE HARM.

◯ Agree
◯ Disagree

Please describe your reasoning for being outside the consensus here.

____

The Delphi panelists found that coverage that only or predominantly includes the perspectives of law enforcement has the potential to cause MODERATE HARM to firearm-injured people and/or co-victims who have been affected by previous shootings.

Please specify if you agree with the Delphi panel's rating of MODERATE HARM.

◯ Agree
◯ Disagree

Please describe your reasoning for being outside the consensus here.

____

The Delphi panelists found that coverage that only or predominantly includes the perspectives of law enforcement has the potential to cause SEVERE HARM to news audiences viewing/reading or listening to the content and/or society at large.

Please specify if you agree with the Delphi panel's rating of SEVERE HARM.

◯ Agree
◯ Disagree

Please describe your reasoning for being outside the consensus here.

____

The following three questions concern potential harm caused by news coverage that includes graphic and/or explicit content about firearm violence (video of a shooting, still photo of a body, audio of screaming).

Please only select disagree if you strongly believe that the average rating of the Delphi panel is incorrect.

The Delphi panelists found that coverage that includes graphic and/or explicit content about firearm violence (video of a shooting, still photo of a body, audio of screaming) has the potential to cause SEVERE HARM to firearm-injured people and/or co-victims involved in the shooting being reported on.

Please specify if you agree with the Delphi panel's rating of SEVERE HARM.

◯ Agree
◯ Disagree

Please describe your reasoning for being outside the consensus here.

____

The Delphi panelists found that coverage that includes graphic and/or explicit content about firearm violence (video of a shooting, still photo of a body, audio of screaming) has the potential to cause SEVERE HARM to firearm-injured people and/or co-victims who have been affected by previous shootings.

Please specify if you agree with the Delphi panel's rating of SEVERE HARM.

◯ Agree
◯ Disagree

Please describe your reasoning for being outside the consensus here.

____

The Delphi panelists found that coverage that includes graphic and/or explicit content about firearm violence (video of a shooting, still photo of a body, audio of screaming) has the potential to cause SEVERE HARM to news audiences viewing/reading or listening to the content and/or society at large.

Please specify if you agree with the Delphi panel's rating of SEVERE HARM.

◯ Agree
◯ Disagree

Please describe your reasoning for being outside the consensus here.

____

The following three questions concern potential harm caused by news coverage that includes the mugshot of the suspected perpetrator of firearm violence.

Please only select disagree if you strongly believe that the average rating of the Delphi panel is incorrect.

The Delphi panelists found that coverage that includes the mugshot of the suspected perpetrator of firearm violence has the potential to cause MODERATE HARM to firearm-injured people and/or co-victims involved in the shooting being reported on.

Please specify if you agree with the Delphi panel's rating of MODERATE HARM.

◯ Agree
◯ Disagree

Please describe your reasoning for being outside the consensus here.

____

The Delphi panelists found that coverage that includes the mugshot of the suspected perpetrator of firearm violence has the potential to cause MODERATE HARM to firearm-injured people and/or co-victims who have been affected by previous shootings.

Please specify if you agree with the Delphi panel's rating of MODERATE HARM.

◯ Agree
◯ Disagree

Please describe your reasoning for being outside the consensus here.

____

The Delphi panelists found that coverage that includes the mugshot of the suspected perpetrator of firearm violence has the potential to cause MODERATE HARM to news audiences viewing/reading or listening to the content and/or society at large.

Please specify if you agree with the Delphi panel's rating of MODERATE HARM.

◯ Agree
◯ Disagree

Please describe your reasoning for being outside the consensus here.

____

Please use this space to add any comments you may have about harmful news content, severity of harm and levels of harm we did not cover in this survey.

____

You may click "Expand" to make the response box longer to fit your answers.

Please use this space to offer any information on harmful news elements on firearm violence that you have not included elsewhere.

____

Congratulations! You have completed the final survey of this Delphi Panel. Thank you so much for your input.<br><br>In order to compensate you for your time, we will need to collect some personal information from you, including your name, date of birth, and address. This information will be kept separate from your survey responses to protect your anonymity. The ClinCard for $75 will be mailed to you after you provide this information. Would you like to proceed?	◯ Yes<br>◯ No

Please click on the following link to provide your information for compensation:

Participant Information for Compensation

As a reminder, your personal information will be kept separate from your survey responses.

Once you have opened this link in a new browser tab, please CLICK SUBMIT BELOW to complete this survey.

Thank you for completing the survey. If you change your mind and would like to receive compensation for your participation, please contact Dr. Jessica Beard at jessica.beard@tuhs.temple.edu.

Please CLICK SUBMIT below when you are done.
