# Supplementary figures and images for: Defining harmful news reporting on community firearm violence: A modified Delphi consensus study
Source: PLoS One. 2024 Dec 18;19(12):e0316026. doi: 10.1371/journal.pone.0316026 (PMC11654925; doi:10.1371/journal.pone.0316026)

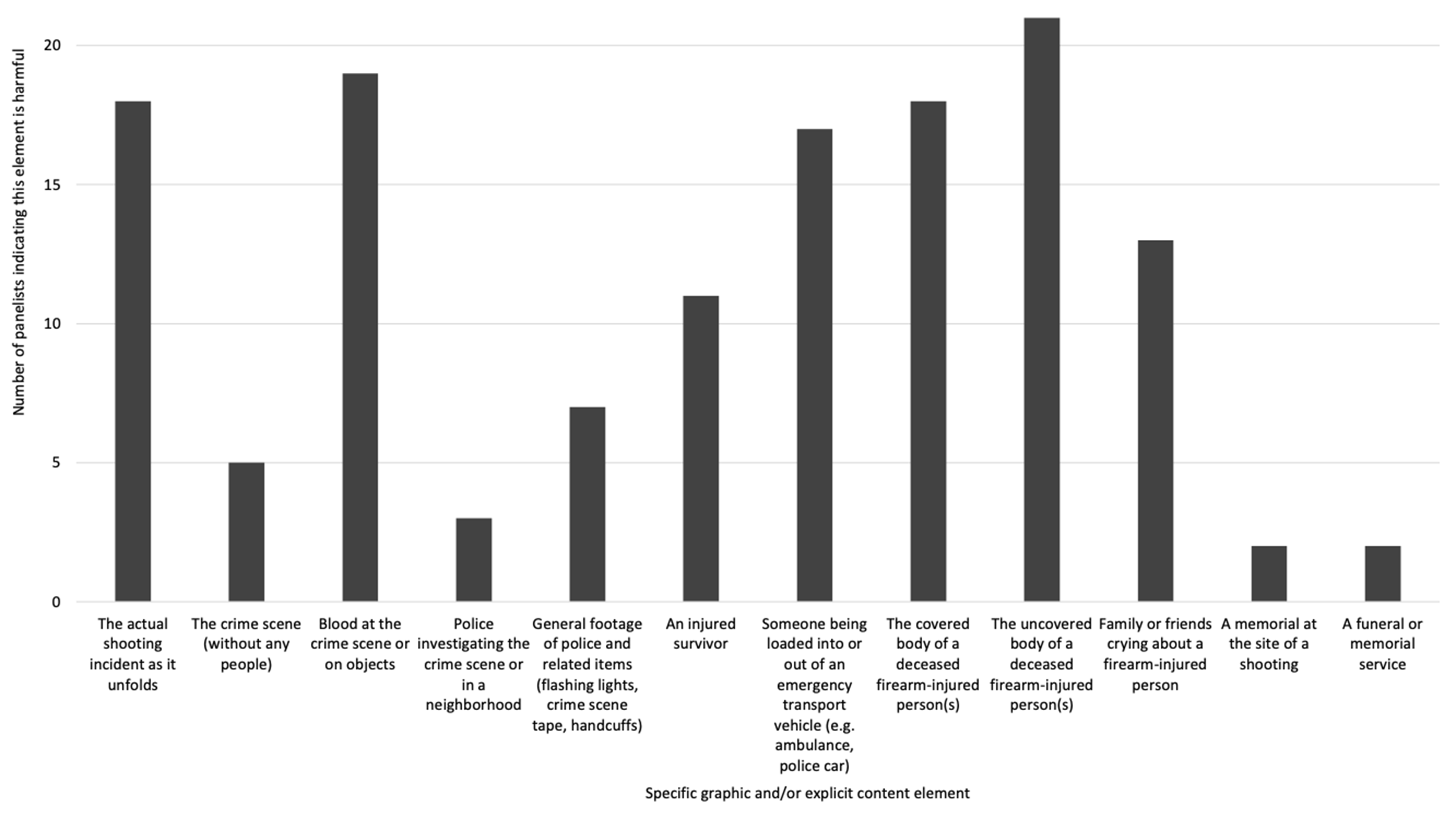

Supplement: S1 Fig — (TIF) [file pone.0316026.s004.tif]
