# Supplementary material for: Defining harmful news reporting on community firearm violence: A modified Delphi consensus study
Source: PLoS One. 2024 Dec 18;19(12):e0316026. doi: 10.1371/journal.pone.0316026 (PMC11654925; doi:10.1371/journal.pone.0316026)
Supplement: S1 Table — (DOCX) [file pone.0316026.s005.docx]

**S1 Table. Median Harmfulness Scores of Formats for Graphic and/or Explicit by Level of Harm and Severity Ratings**

| **Element** | **Median Harmfulness Score (Rating)^a^** | | |
| --- | --- | --- | --- |
|  | Individual^b^ | Community^c^ | Society^d^ |
| Disturbing video | 9 (Severe) | 9 (Severe) | 8 (Severe) |
| Disturbing still images | 9 (Severe) | 9 (Severe) | 8 (Severe) |
| Disturbing audio | 8 (Severe) | 9 (Severe) | 8 (Severe) |
| Disturbing detailed verbal description | 8 (Severe) | 8 (Severe) | 7 (Moderate) |

*^a^*Severity ratings are: Severe, 10-8; Moderate, 7-5; and Mild, 4 and below.

^b^Individual defined as: Firearm-injured people and/or co-victims involved in the shooting being reported on.

^c^Community defined as: Firearm-injured people and/or co-victims who have been affected by previous shootings.

^d^Society defined as: News audiences viewing, reading, and/or listening to the content and/or society at large.
