# Supplementary material for: Defining harmful news reporting on community firearm violence: A modified Delphi consensus study
Source: PLoS One. 2024 Dec 18;19(12):e0316026. doi: 10.1371/journal.pone.0316026 (PMC11654925; doi:10.1371/journal.pone.0316026)
Supplement: Attachment — Submitted filename: Response to Reviewers.docx [file pone.0316026.s006.docx]

**Response to Reviewers**

**Title:** Defining Harmful News Reporting on Community Firearm Violence: A Modified Delphi Consensus Study

Thank you for the opportunity to revise our manuscript entitled “Defining Harmful News Reporting on Community Firearm Violence: A Modified Delphi Consensus Study.” We appreciate the editor’s and reviewers’ thoughtful comments and believe they have substantially strengthened the manuscript. We have revised our manuscript to address the comments and suggestions by the editor and reviewers as follows:

**Comments from the Editor:**

*1. Please ensure that your manuscript meets PLOS ONE's style requirements, including those for file naming.*

**The file is now uploaded following PLOS ONE’s style requirements.**

*2. PLOS requires an ORCID iD for the corresponding author in Editorial Manager on papers submitted after December 6th, 2016. Please ensure that you have an ORCID iD and that it is validated in Editorial Manager.*

**The ORCID iD for Dr. Beard, the corresponding author, is 0000-0002-3844-864X. We still still have been unable to validate it in Editorial manager. There is currently a ticket in with the *PLOS One* Journal Office to try and resolve this issue.**

*3. Thank you for stating the following financial disclosure: "This research was funded by the Stoneleigh Foundation (https://stoneleighfoundation.org/) (JHB) and the National Institute on Minority Health and Health Disparities of the National Institutes of Health (JHB, CNM, JM) [grant number R21MD019088, https://www.nimhd.nih.gov/]. ELE is supported by the National Institute on Drug Abuse of the NIH [grant number T32DA031099, https://nida.nih.gov/]. The content is solely the responsibility of the authors and does not necessarily represent the official views of the Stoneleigh Foundation or the NIH." Please state what role the funders took in the study.  If the funders had no role, please state: ""The funders had no role in study design, data collection and analysis, decision to publish, or preparation of the manuscript."" If this statement is not correct you must amend it as needed. Please include this amended Role of Funder statement in your cover letter; we will change the online submission form on your behalf.*

**We have included the text indicating that the funders had no role in the study in the cover letter for this resubmission.**

*4. Thank you for stating the following in the Acknowledgments Section of your manuscript: "This research was funded by the Stoneleigh Foundation (JHB) and the National Institute on Minority Health and Health Disparities of the National Institutes of Health (JHB, CNM,JM) [grant number R21MD019088]. ELE is supported by the National Institute on Drug Abuse of the NIH [grant number T32DA031099]. The content is solely the responsibility of the authors and does not necessarily represent the official views of the Stoneleigh Foundation or the NIH." We note that you have provided funding information that is not currently declared in your Funding Statement. However, funding information should not appear in the Acknowledgments section or other areas of your manuscript. We will only publish funding information present in the Funding Statement section of the online submission form. Please remove any funding-related text from the manuscript and let us know how you would like to update your Funding Statement. Currently, your Funding Statement reads as follows: "This research was funded by the Stoneleigh Foundation (https://stoneleighfoundation.org/) (JHB) and the National Institute on Minority Health and Health Disparities of the National Institutes of Health (JHB, CNM, JM) [grant number R21MD019088,https://www.nimhd.nih.gov/]. ELE is supported by the National Institute on Drug Abuse of the NIH [grant number T32DA031099, https://nida.nih.gov/]. The content is solely the responsibility of the authors and dose not necessarily represent the official views of the Stoneleigh Foundation or the NIH." Please include your amended statements within your cover letter; we will change the online submission form on your behalf.*

**We have removed the funding-related text from the manuscript and included it—along with the text indicating that the funders had no role in the study—in the cover letter for this resubmission.**

*5. In the online submission form, you indicated that data cannot be shared publicly to protect participant anonymity. Data are available upon request from the corresponding author for researchers who meet the criteria for access to confidential data. All PLOS journals now require all data underlying the findings described in their manuscript to be freely available to other researchers, either 1. In a public repository, 2. Within the manuscript itself, or 3. Uploaded as supplementary information. This policy applies to all data except where public deposition would breach compliance with the protocol approved by your research ethics board. If your data cannot be made publicly available for ethical or legal reasons (e.g., public availability would compromise patient privacy), please explain your reasons on resubmission and your exemption request will be escalated for approval.*

**Thank you for the opportunity to clarify this point. Because our research includes a small panel of experts, and the methodology relies on anonymity to encourage equal participation by the panelists, we are not able to share our data publicly. If we did share the data, we are concerned that participants could be identifiable, which could place their privacy at risk. Keeping this data completely confidential and not sharing it publicly was stipulated both in our ethical review and in the terms of our grant from the NIH. We are happy to provide more information if needed.**

**Comments from Reviewer #1:**

*1. This paper raises some very important points about the role that media reporting can play in furthering the harms that are caused by firearm violence in communities. It is especially good to see attention being given to the disproportionate role that police sources play in shaping how news is framed. However, there is room for some improvement.*

**Thank you for acknowledging the contributions of this study and for the thoughtful and helpful comments in your review.**

*2. The paper currently lacks nuance, and makes many statements that draw artificial binaries – for example, that gun violence is framed as a crime issue when it should be seen as a public health issue. In reality, it is both of these, and far more – issues such as historical racism and its contemporary consequences, severe poverty, and socioeconomic marginalization go far beyond crime or public health. I would like to see a much more sophisticated theoretical and empirical perspective that takes this complexity into account.*

**Thank you for drawing attention to ways in which we could better communicate our perspective on community firearm violence and news framing. We completely agree that community firearm violence is a highly complex issue rooted in historical and contemporary racism, which we outline in the introductory paragraphs of this paper (Page 3). To address the concern you raise between community firearm violence being framed simplistically as a crime issue versus a public health issue, we have reworded and added explanation to the Introduction (Pages 3-4) to more clearly state that: 1) crime framing is specifically mentioned because of decades of journalism and communication research that clearly shows the dominance and harm of crime framing in news reports, and 2) compared to crime framing, public health framing is one example of framing that may allow for greater attention to be paid to these underlying, fundamental causes of community firearm violence. To prevent this from appearing to be a simple binary, we have also added the example of Solutions Journalism as another recommended approach journalists can take to tell impactful stories about social problems.**

*3. Given that the entire purpose of the paper is to define ‘harmful’ reporting, it would be helpful to see more attention given to discussing current conceptualizations of, and evidence for, harm (or the lack of conceptualizations/evidence) and exploring how/why they differ or are unsatisfactory for research purposes.*

**We now include a more explicit discussion of recent conceptualizations of harmful reporting to further contextualize this study and its potential contributions. Under the subheading of “Defining harmful reporting as a next step for intervention” in the introduction (Page 5), we now provide readers with a paragraph of context about harmful reporting related to other pressing health issues (e.g. suicide, mass shootings, sexual assault, abuse, and crime involving minors), with a focus on harmful reporting related to suicide. We believe this helps provide comparative context to the current state of evidence on harmful reporting related to CFV—which is much less developed—as well as what the research-to-practice-to-impact trajectory may look like for this study’s efforts to reach expert consensus on harmful reporting for CFV.**

*4. Further, there is a need to better explain whether and how the different ‘levels’ of harm were conceptualized/what guidelines were used to attempt to ensure some consistency between different individuals providing ratings – for instance, how can we be sure that different people in the different groups were using the same ‘yardstick’ to label something as an extreme level of harm? The results may only be saying that some people/groups see things as more harmful than others – rather than telling us anything about actual harm caused.*

**Thank you for this comment. It is certainly likely that there is some degree of variation between panelists’ idea of what constitutes various numeric values, as is the case with any Likert-like scale. To minimize this variation, we followed best practices for the use of Likert-like scales, including providing the same clear, standardized instructions to all panelists and providing value labels at intermediate values. We now mention these steps taken in the manuscript, stating: “We asked panelists to rate the severity of potential harm for each news content element for each of the three levels of harm on an 11-point scale of 0 to 10. We provided the same detailed instructions to all panelists and provided descriptive labels on intermediate values (i.e., 0 indicated *no harm*, 5 indicated *some harm*, and 10 indicated *extreme harm*).” (Page 11)

Moreover, we are indeed measuring perceptions of harm rather than assessing more objective measures of harm (e.g., health outcomes), as the Delphi method is meant to achieve expert consensus on subjective matters. We have now reinforced this point twice, once where we now state, “However, it is important to note that this study did not measure the direct impact of harmful reporting on individuals, impacted communities, or society, which remain important directions in future research” (Page 20) and in the newly expanded limitations section, where we now state, “In this study, the aim was to achieve expert consensus on what constitutes harmful reporting on firearm violence, which inherently relies on the perceptions and perspectives of the panelists. As such, we did not seek to measure the direct impact of harmful reporting on health or psychological outcomes, which will be an important area of research going forward.” (Page 22)**

*5. This is particularly problematic in the context of lived experience participants, whose assessments could be influenced by a wide range of factors including trauma. This is touched on in the discussion, but only in the context of trauma-informed reporting (which means what, exactly?). The paper needs to be very careful about differentiating between subjective perceptions of harm, and whether those perceptions translate to harm in more ‘objective’ terms (for example, a person may believe that media coverage causes “narrow mindedness” – but is there anything to back this belief up?).*

**Thank you for raising the very important issue of trauma. It is true that people with lived experience frequently face trauma from their experiences with community firearm violence; this was extremely clear in the qualitative work we have done from which this present study draws. It is also an empirical question whether panelists with lived experience expertise report on average differently. To address this, we report on statistical tests of differences in reporting between expertise groups throughout the paper to note when this may be this case (Page 13, Page 15, Page 19).

In response to your question, we also now define and provide sources for trauma-informed reporting, stating: “Trauma-informed journalism employs methods that aim to recognize and respond effectively to the experiences of trauma survivors who are story sources or potential audience members with the goal to minimize further harm that the reporting may cause in the aftermath of a traumatic event (65-67) (Page 20).

Further, as we mention in our response to your previous comment above, we are now much more explicit about how the Delphi method we used is in fact ultimately measuring perceived harm as informed by these panelists’ various types of expertise in the matter.**

*6. I am a little concerned about the overall objectivity and relative lack of serious critical analysis presented in the work. For example, something that is not really acknowledged is that, at times, an element that may be deemed ‘harmful’ may nevertheless be an important part of a balanced, objective, and accurate news story.*

**In this paper, we present and contextualize the results of a modified Delphi consensus method, which is a rigorous and well-established method for reaching expert consensus on often complex and sometimes divisive issues. We have added more description on this methodology on Page 8. This approach was selected, as we now state, because it “provided the opportunity to bring diverse experts into nuanced conversation that would not be achieved through a traditional survey or other quantitative research methods.” This method is not without its limitations, however, and we now explicitly present many more of these limitations in our revised limitations section (Page 22).**

**We also see how it is possible that something that the panelists in this study deemed harmful may be included as part of a news report. Ultimately, it comes down to the choices of journalists—who are trained to do exactly this—to balance the potential harms of these elements versus their potential probative value. This study was not meant to develop a list of things that should not be included, but instead to further advance research on this topic to inform guidelines and best practices to minimize any harms of reporting, which will inherently be iterative and evolve over time. We have now included statements to this effect, stating: “This study provides an important starting point from which to build scholarship around harmful reporting on firearm violence, work which will surely be iterative and evolve over time. The findings of this study will inform future investigations measuring the frequency and severity of harmful reporting on CFV in news reports, the direct impacts of harmful reporting on individuals and communities, along with interventions with journalists to minimize harmful reporting on CFV” (Page 23).**

*7. Similarly, some aspects that are implicitly considered to reduce harm – such as ‘providing solutions’ – may cause harm in their own right (e.g., ineffective ‘solutions’ may be promoted). Again, this speaks to the need for greater nuance and depth in how this paper is written.*

**This is a very valid point that certainly merits explicit mention in the discussion. We have now added the following text to highlight how the decision to include “solutions” should also be well-reasoned and consider the evidence base behind said solutions: “Finally, this study did not evaluate the news content elements that should be present to support less harmful reporting, as there is far less existing research in this area. Thus, our results should not be interpreted to suggest that inclusion of firearm violence solutions is by definition ‘helpful,’ as news reporting about ineffective solutions without a critical lens could certainly cause harm.” (Page 22)**

*8. I am concerned, in relation to the lived experience participants in particular, that the small sample size is unlikely to capture the breadth of different experiences people may have with media reporting.*

**As we now explain in our additions detailing the Delphi method, the goal of this method is to achieve consensus among experts. Our inclusion of lived experience expert panelists helps address the multiple types of expertise that one may have around this issue, and we based our elements presented in Round 1 on qualitative research with people who survived a firearm injury. We have changed the word participant throughout the paper to panelist, to emphasize that the experts included in this study are not meant to encompass the breadth of experience but instead present and use their specific expertise. On Page 22, we do mention that including lived experience experts beyond Philadelphia may have added depth to our survey responses.**

*9. The over-representation of female participants is also a limitation.*

**This was not included as a limitation because the gender of experts is not relevant to the validity of their expertise.**

*10. Overall, the discussion takes an uncritical approach to the findings and accepts, seemingly without question, that the findings are valid, robust, and should be acted on. While clearly well intentioned, this comes across more as activism than scholarship. The paper also needs to be far more open about the limitations of the work.*

**Thank you for your helpful comments in this review, especially the encouragement to be more explicit about the method of this study and its limitations. We have added a much more detailed description of the Delphi method and have added extensively to our mentioned limitations. Specifically, we have emphasized that the study is not measuring the direct effect of harmful reporting as you have suggested.**

**Comments from Reviewer #2:**

*1. Insightful and much needed article on harmful news content and gun violence reporting. I believe this manuscript will make significant contributions to our knowledge on the ways media content specifically gun violence is framed and in what ways we can collectively address harmful content. Overall the paper is well written. However, I do have several minor revisions.*

**Thank you for recognizing the importance of this article and the insights it adds to the field, as well as for the revisions you request below.**

*2. Author(s) should explain in more detail the Delphi method and why did the authors choose this approach.*

**We now have added additional information on the Delphi method and why it was chosen for this study (Page 8). We also now refer to the rounds as “rounds” rather than surveys and the participants as “panelists” to emphasize key aspects of the Delphi method.**

*3. In regard to the recruitment of the sample the author(s) did not address: How were participants invited?*

**We state that panelists were identified by “working with PCGVR [Philadelphia Center for Gun Violence Reporting] leadership to identify key stakeholders from three distinct expertise backgrounds,” and that panelists were “contacted over email” (Pages 7-8).**

*4. If I am reading this correctly, a total of six participants with lived experience participated in study. What were their demographics? I am concerned with merging the sub-groups to arrive at a median age because most victims of violence are younger than 48. What is the average age of a gun violence survivor in Philadelphia this should be reflective in the age of the lived experience group. Furthermore, this sub-group should have been disproportionately over-represented than the other sub-groups in the study. The sample should be more reflective of people injured by firearm Philadelphia. If one of the aims is to center the voices of those most impacted by gun violence (which was evident by one of the categories), then the community voices should be over-represented in sample instead there was far more representation of journalists and scholars. This is a limitation of the study that should be acknowledged.*

**Thank you for seeking further clarification on the lived experience experts in this panel. We now make it clearer that we include both people who have been shot as well as loved ones of people who have been shot (i.e., “co-victims”) in our lived experience experts group. This means that it is possible that this group would in fact have a higher median age than people sustained gunshot wounds in Philadelphia, because co-victims may be parents and other loved ones, which was the case in this study. We do not present stratified demographics in this study to protect panelist anonymity, but this group did have the widest range of ages, with the youngest participant in the 18-21 age range.**

**We also agree about the importance of representation of people with lived experience in research. The Round 1 content was inspired by the results of our qualitative research study with 26 people with lived experience (e.g. participants were recently firearm-injured/shot). In this way, though the Delphi panelists were approximately evenly split between the three expertise groups, those with lived experience’s perspectives were centered from the onset of the study. This centering of the qualitative study results is emphasized on Page 9 now: “This approach ensured that the perspectives of people with lived experience of CFV were prioritized in the development of content for Round 1 (42).”**

*5. Author(s) should also define: What does lived experience mean? Did participants witness violence, were they victims and/or perpetrators of violence?*

**Thank you for the opportunity to clarify this point. For this study, lived experience means both people who have been shot and survived as well as loved ones of firearm-injured people (“co-victims”). We now provide a clearer definition of lived experience when we first mention lived experience expertise: “(1) lived experience experts (people who have been shot and survived as well as co-victims, who are the loved ones of firearm-injured people)” (Page 7).**
